# Pharmacogenetic & Pharmacokinetic Biomarker for Efavirenz Based ARV and Rifampicin Based Anti-TB Drug Induced Liver Injury in TB-HIV Infected Patients

**DOI:** 10.1371/journal.pone.0027810

**Published:** 2011-12-06

**Authors:** Getnet Yimer, Nobuhisa Ueda, Abiy Habtewold, Wondwossen Amogne, Akira Suda, Klaus-Dieter Riedel, Jürgen Burhenne, Getachew Aderaye, Lars Lindquist, Eyasu Makonnen, Eleni Aklillu

**Affiliations:** 1 Division of Clinical Pharmacology, Department of Laboratory Medicine, Karolinska Institutet, Stockholm, Sweden; 2 Department of Pharmacology, Faculty of Medicine, Addis Ababa University, Addis Ababa, Ethiopia; 3 Department of Internal Medicine, Faculty of Medicine, Addis Ababa University, Addis Ababa, Ethiopia; 4 Department of Medicine, Division of Infectious Diseases, Karolinska Institutet, Stockholm, Sweden; 5 Department of Clinical Pharmacology and Pharmacoepidemiology, University of Heidelberg, Heidelberg, Germany; Dr. Margarete Fischer-Bosch Institute of Clinical Pharmacology, Germany

## Abstract

**Background:**

Implication of pharmacogenetic variations and efavirenz pharmacokinetics in concomitant efavirenz based antiviral therapy and anti-tubercular drug induced liver injury (DILI) has not been yet studied. We performed a prospective case-control association study to identify the incidence, pharmacogenetic, pharmacokinetic and biochemical predictors for anti-tubercular and antiretroviral drugs induced liver injury (DILI) in HIV and tuberculosis (TB) co-infected patients.

**Methods and Findings:**

Newly diagnosed treatment naïve TB-HIV co-infected patients (n = 353) were enrolled to receive efavirenz based ART and rifampicin based anti-TB therapy, and assessed clinically and biochemically for DILI up to 56 weeks. Quantification of plasma efavirenz and 8-hydroxyefaviernz levels and genotyping for *NAT2*, *CYP2B6*, *CYP3A5*, *ABCB1*, *UGT2B7* and *SLCO1B1* genes were done. The incidence of DILI and identification of predictors was evaluated using survival analysis and the Cox Proportional Hazards Model. The incidence of DILI was 30.0%, or 14.5 per 1000 person-week, and that of severe was 18.4%, or 7.49 per 1000 person-week. A statistically significant association of DILI with being of the female sex (p = 0.001), higher plasma efavirenz level (p = 0.009), efavirenz/8-hydroxyefavirenz ratio (p = 0.036), baseline AST (p = 0.022), ALT (p = 0.014), lower hemoglobin (p = 0.008), and serum albumin (p = 0.007), NAT2 slow-acetylator genotype (p = 0.039) and *ABCB1 3435TT* genotype (p = 0.001).

**Conclusion:**

We report high incidence of anti-tubercular and antiretroviral DILI in Ethiopian patients. Between patient variability in systemic efavirenz exposure and pharmacogenetic variations in *NAT2*, *CYP2B6* and *ABCB1* genes determines susceptibility to DILI in TB-HIV co-infected patients. Close monitoring of plasma efavirenz level and liver enzymes during early therapy and/or genotyping practice in HIV clinics is recommended for early identification of patients at risk of DILI.

## Introduction

Tuberculosis (TB) is the most common opportunistic fatal infection in HIV infected patients [1]. Concomitant HIV and TB treatment is recommended in patients with low CD4 cell counts. Although effective therapy is available for both TB and HIV, concurrent treatment is complicated due to adverse drug reactions [2]. Anti-tuberculosis and antiretroviral drug induced liver injury (DILI), a common serious adverse drug reaction, is one of the most challenging clinical problems, cause of hospitalization and life-threatening events [3], [4]. DILI can be fatal if therapy is not interrupted on time, and the subsequent adherence problem may cause treatment failure and, relapse or drug resistance [5]–[7]. The reported incidence of DILI during TB treatment varies from 5 to 33% [5], [6]. In patients receiving anti-retroviral therapy between 14–20% experience elevations of liver enzymes, and about 2–10% need to interrupt anti-retroviral therapy due to severe hepatic injury and marked elevation of liver enzymes [8], [9].

Among the first-line TB drugs pyrazinamide, isoniazid and rifampicin have all been associated with DILI [2]. All classes of antiretroviral drugs are associated with potential risk of DILI, though higher incidence has been noted for nevirapine, efavirenz and boosted PIs [10], [11]. Concomitant anti-TB and ARV therapy exacerbates risk for DILI [12], [13], and overlapping toxicity between drugs used to treat HIV and tuberculosis could also complicate the management. Although rifampicin (RIF) and efavirenz (EFV) are key drugs used for concomitant TB and HIV therapy in resource limited settings, data on the concomitant use related liver injury and biomarkers are limited particularly from Sub-Saharan Africa, a continent highly affected by HIV/AIDS and tuberculosis [14].

Metabolic pathway of efavirenz is complex as being a substrate inducer and/or inhibitor of its own metabolism involving several CYP enzymes with varying activities [15], [16]. It is primarily biotransformed to 8-hydroxyefavirenz mainly by *CYP2B6*, to a minor extent by *CYP3A* [15], [17]. Efavirenz and its primary and secondary metabolites undergo conjugation mainly via *UGT2B7* [17], [18]. Although there are conflicting suggestions as to whether efavirenz is a substrate for P-glycoprotein we and others have previously reported the effect of *ABCB1* genetic variation on efavirenz pharmacokinetics [19]–[21]. NAT2 is the main enzyme responsible for metabolism of isoniazid [22] and association of its genetic polymorphism with isoniazid induced liver injury has been a subject of exploration [23]–[25]. Organic anion-transporting polypeptide *OATP1B1*, coded by *SLCO1B1*, is a liver-specific uptake transporter important in hepatic drug disposition. RIF is both substrate and inhibitor of *OATP1B1* [26]. All these enzymes and transporter proteins are genetically polymorphic and inducible by several drugs. Both RIF and efavirenz induces *CYP2B6*, *CYP3A4*, *UGT2B7*, *ABCB1* and *SLCO1B1* [27]–[30]. Induction can lead to drug–drug interactions and decreased exposure in the liver and/or increased toxic metabolite formation. Interaction could be modified by other anti-tubercular agents such as isoniazid which inhibits many cytochrome P450 enzymes including *CYP3A* [31] and may counter balance the inducing effect of RIF [32].

Plasma concentrations of HIV and TB drugs display wide inter-individual variability partly due to genetic variations in the respective drug metabolizing enzymes or transporter proteins. Genetic variation is believed to play an important role in DILI [23]–[25]. Pharmacogenetic studies of DILI is focused on the formation of toxic and immunogenic drug metabolites, hepatobiliary transporters and drug metabolizing enzymes [23]–[25]. We recently reported incidence as well as pharmacogenetic and pharmacokinetic predictors of efavirenz based ART induced liver injury in HIV only infected patients [33]. In the present study we investigated incidence and predictors of concomitant efavirenz based ART and rifampicin based anti-tuberculosis drugs induced liver injury in HIV-TB co-infected patients. The present study was designed based on the following hypotheses: i) the use of anti-tuberculosis drugs (mainly rifampicin and isoniazid) as well as ARV drugs is associated with liver injury. Hence concomitant uses of these drugs exacerbate the incidence of DILI, ii) anti-tuberculosis drugs mainly rifampicin induces *CYP2B6* and *CYP3A5* [27], [28] lowering efavirenz plasma concentration while isoniazid inhibits some CYP enzymes [34]. Because of drug-drug interactions between anti-tuberculosis and ARV drugs, concomitant use these drugs may present with modified genetic and kinetic biomarkers than when used separately. Effect of genetic variations on drug metabolizing enzymes and hepatocellular transporters may be altered in the presence of inducers. Hence identified risk factors for anti-tubercular or ARV therapy alone may not represent the finding from concomitant TB-HIV therapy. To our knowledge, no pharmacogenetic and efavirenz kinetics association studies with respect to concomitant efavirenz based ART and rifampicin based anti-TB DILI have been reported previously. In the present study we performed prospective comprehensive case-control association study for efavirenz based antiretroviral and RIF based anti-TB DILI in HIV-TB co-infected in Ethiopia, the second densely populated country in Africa with 1.5 million HIV infected individuals and ranks 7^th^ in the WHO high TB-burden country list. Biochemical variables, efavirenz pharmacokinetics as well as pharmacogenetic variations in six candidate genes relevant for metabolism and transport of ARV and anti-TB drugs; namely; *CYP2B6*, *CYP3A5*, *UGT2B7*, *NAT2*, *ABCB1* and *SLCO1B1* (*OATP1B1*) were investigated.

## Methods

### Ethics Statement

The study protocol was approved by the Regional Ethical Review Board in Stockholm at Karolinska Institutet, Sweden; Institutional Review Board (Faculty. Research and Publication Committee) at Faculty of Medicine, Addis Ababa University; The National Ethics Review Committee at Ethiopian Science and technology Ministry as well as by the Food and Drug Administration and Control Authority of Ethiopia. Written informed consent was obtained from each subject before the start of this study.

### Study participants

Newly diagnosed ART and anti-TB treatment naïve adult TB and HIV co-infected patients (n = 373) were recruited and enrolled prospectively and followed up to one year during June 2007 to January 2011. The eligibility criteria were age ≥18 years, CD4 count <200 cells/UL, not pregnant and not on other known hepatotoxic drugs concurrently (except co-trimoxazole, 960 mg per day, which was given for all participants before enrolment and during the follow up period according to the treatment guideline). None of the participants received isoniazid prophylaxis and treatment for tuberculosis two years before enrolment and during the study period.

### Treatment, clinical and laboratory investigations

All study participants received RIF based short-course chemotherapy for TB following the national TB treatment guideline. ART was then initiated with 600 mg efavirenz based HAART containing stavudine/lamivudine/efavirenz (D4T/3TC/EFV) or zidovudine/lamivudine/efavirenz (AZT/3TC/EFV) or tenofovir/lamivudine/efavirenz (TDF/3TC/EFV). A complete history and physical examination were taken before enrolment and at the scheduled and unscheduled visits. Laboratory tests were performed before anti TB initiation included complete and differential blood counts, platelet count, CD4 count, HIV RNA determination, hepatitis B surface antigen, anti-hepatitis C antibody, serum albumin, renal function tests, liver tests including; aspartate aminotransferase (AST), alanine aminotransferase (ALT), alkaline phosphatase (ALP), and direct and total bilirubin. Follow-up for liver enzymes were performed at before and on the 1^st^, 2^nd^, 4^th^, 8^th^, 12^th^, 24^th^, 48^th^, and 56^th^ weeks after initiation of anti-TB treatment.

### CYP2B6, CYP3A5 UGT2B7, ABCB1, UGT2B7 and SLCO1B1 genotyping

Blood sample was obtained from 201 patients for genotype analysis. Genomic DNA was isolated from peripheral blood leukocytes using QIAamp DNA Maxi Kit (QIAGEN GmbH. Hilden. Germany). Genotyping was carried out at the division of clinical pharmacology, Department of laboratory medicine. Karolinska University Hospital-Huddinge, Karolinska Institute Stockholm, Sweden.

Allelic discrimination reactions were performed using TaqMan® (Applied Biosystems, CA, USA) genotyping assays: (C__7586657_20 for *ABCB1* 3435C>T rs1045642, C__11711730_20 for *CYP2B6* 516G>T rs3745274 [CYP2B6*6] , C__30720663_20 for *UGT2B7*-372G>A rs7662029 [UGT2B7*2b,*2c,*2d,*2f], C__26201809_30 for *CYP3A5* 6986A>G rs776746 [CYP3A5*3], C__30203950_10 for *CYP3A5* 14690G>A g.14690G>A [CYP3A5*6], C__32287188_10 for *CYP3A5* g.27131_27132insT rs241303343 [CYP3A5*7], C___1901697_20 for SLCO1B1 388A>G rs2306283 (*1b) and C__30633906_10 for SLCO1B1 521T>C rs4149056 (*5) on ABI 7500 FAST (Applied Biosystems, Foster City, CA). The final volume for each reaction was 10 µl, consisting of 2× TaqMan Universal PCR Master Mix® (Applied Biosystems), 20× drug metabolising genotype assay mix and 10 ng genomic DNA. The PCR profile consisted of an initial step at 50°C for 2 min and 50 cycles with 95°C for 10 minutes and 92°C for 15 sec.

### NAT2 gene sequencing

The coding regions of NAT2 gene was amplified by PCR using a forward primer (5′- GTCACACGAGGAAATCAAATGC-3′) and a reverse primer (5′-GTTTTCTAGCATGAATCACTCTGC-3′) as described previously.[35], [36] The PCR products were purified using ExoSAP-IT® (USB Corporation, Cleveland, OH) PCR Purification kit. Sequencing was done in forward and reverse directions using PCR primers described above and an internal reverse primer (5′-GGATGAAAGTATTTGATGTTTAGG-3′). Sequencing reaction was done using the ABI PRISM™ BigDye® terminator cycle sequencing ready reaction kit v3.1 (Applied Biosystems, Foster City, CA), and analyzed on an ABI Prism 377 DNA sequencer. *NAT2* sequence chromatograms were visually inspected and analyzed using software program FinchTV version.1.4.0 (htpp://www.geospiza.com) and aligned with the *NAT2* reference sequence (http://www.ncbi.nlm.nih.gov; GenBank reference: NM 000015.2) for SNPs identification.

### Quantification of plasma efavirenz and 8-hydroxyefavirenz concentration

On the 4^th^ week of initiation of efavirenz -based HAART, 8 ml of blood samples were collected 16 hrs post efavirenz dosing in a vacutainer CPT (Becton Dickinson, Heidelberg, Germany) from 212 patients, centrifuged (1700 g for 20 min), and 2 mL plasma aliquot was stored at −80°C for determination of efavirenz and its metabolite concentrations. Plasma samples were sent in dry ice to the Department of Clinical Pharmacology and Pharmacoepidemiology, University of Heidelberg. Germany. Plasma efavirenz and 8-hydroxyefavirenz concentration were determined by LC/MS/MS as described previously [37]. The lower limits of quantification in plasma were 10.0 ng/mL for efavirenz and 0.4 ng/mL for 8-hydroxyefavirenz. The efavirenz (8-hydroxyefavirenz) calibration range was 10–10000 ng/mL (0.4–400 ng/mL). Linear regression with 1/× weighing resulted in correlation coefficients of r^2^>0.99. Accuracy and precision (within-batch and batch-to-batch) of the assay fulfilled all recommendations of FDA guidelines.

### Case identification

Patients with DILI were identified according to the CIOMS (Council for International Organizations of Medical Science) criteria, which are based on selected laboratory liver parameters (CIOMS laboratory criteria and the exclusion of any disease-related causes of liver injury) [38]. Liver biochemical parameters more than two times the upper normal limit (UNL) value was considered as DILI. Those ≥5× UNL or equal to threefold elevation in ALT and simultaneous elevation of total bilirubin concentration ≥2× UNL were considered as severe DILI.

### Statistical analysis

Baseline demographic and laboratory parameters were described as median and interquartile range (IQR) for continuous variables and as percentages for categorical variables. Chi-square test was used to compare the observed and expected allele frequencies according to Hardy-Weinberg equilibrium. Haplotype analysis was done using Haploview v.4.1 software. The efavirenz metabolic ratio (EFV MR) was calculated by dividing concentrations of efavirenz by 8-hydroxyefavirenz. Normality of kinetic data was assured by transforming the data to Log 10 values before statistical analysis. Independent t-test was used to compare log transformed plasma efavirenz, 8-hydroxyefavirenz and efavirenz/8-hydroxyefvarinz ratio between patients with and without DILI. Univariate and multivariate Cox proportional Hazards Model were performed to identify potential predictors of DILI and Kaplan-Meier curves was performed to estimate the incidence of DILI over time. Incidence of DILI was calculated as the number of episodes per 1000 persons exposed per week. SPSS version 19.0 for complete data analysis and Statistica version 10 for graphical data presentation were employed. Variables with p<0.05 were considered potential predictors for DILI.

## Results

A total of 373 newly diagnosed antiretroviral and antitubercular treatment naïve HIV-TB co-infected patients among which 199 (53.4%) women and 174 (46.6%) men were enrolled prospectively and followed up for development of DILI for up to 56 weeks after initiation of antiTB treatment. Twenty patients (20.4%) were excluded from the analysis and put on standard treatment as per the national guideline for they had elevated transaminases (>5×UNL) before starting efavirenz therapy. The remaining 353 patients were initiated with the four fixed dose combination anti TB drugs namely rifampicin, ethambutol, pyrazianmide and isoniazid for the intensive phase (2-month) followed with isoniazid and rifampicin for the continuation phase (4-month). All patients were also initiated with either AZT/3TC/EFV or D4T/3TC/EFV or TDF/3TC/EFV within the first 8 weeks after starting anti-TB treatment.

### Baseline characteristics of patients

The median age of participants was 35 years (range 18–72) and 49.2% of them had a BMI of <18.5. Seventy eight patients were diagnosed smear positive TB; while 62, 36, and 177 smear negative, disseminated and extra pulmonary TB, respectively. Screening of participants for Hepatitis B and C showed that 33 (9.3%) were positive for Hepatitis B surface antigen, while 5 (1.4%) was positive for Hepatitis C virus antibody. Association of socio demographic parameters, sex and type of HAART with DILI using Cox-regression analysis is presented in Table 1. Association of DILI with female sex (p = 0.001) and lower BMI (p = 0.09) was noted.

**Table 1:** Association of socio demographic parameters of study participants and type of therapy with DILI.

	*Efavirenz-based ART DILI*		*Hazard ratio (95%CI)*	*p-value*
	Cases = 65)	Cases = 65)		
Sex				0.001
Female (%)	39 (60.0)	148 (51.4)		
Male (%)	26 (40.0)	140 (58.6)	0.43 (0.26–0.71)	
Body mass index				0.09
< = 18.5 (%)	37 (56.3)	132 (45.8)		
>18.5 (%)	28 (43.8)	156 (54.2)	0.66 (0.40–1.07)	
Hepatitis C virus antibody				0.49
Positive (%)	1 (1.5.0)	4 (1.4%)		
Negative (%)	64 (98.5)	284 (98.6)	0.68 (0.62–0.73)	
Hepatitis B surface antigen				0.79
Positive (%)	5 (7.7)	28 (9.7)		
Negative (%)	60 (92.3)	260 (90.7)	1.1 (0.46–2.76)	
Marital status				0.51
Married (%)	21 (32.3)	106 (36.8)		
Divorced (%)	11 (16.7)	43 (14.9)		
Single (%)	22 (34.4)	107 (37.3)		
Widowed (%)	11 (16.7	31 (10.9)		
Type of HAART				0.69
D4T30/3TC/EFV (%)	26 (40.4)	98 (34.1)		
CBV/EFV (%)	23 (34.8)	114 (39.6)		
TDF/3TC/EFV (%)	16 (24.8)	76 (26.4)		

### Incidence and timing for ART and anti-TB DILI

From a total of 353 patients, 106 (30.0%) or 14.5 per 1000 person-week developed DILI. The median time for development of DILI was 2 weeks and the majority (91.6%) of the DILI occurred during the first 8 weeks. Severe DILI, i.e., elevation of transaminases >5 times the upper normal limit was observed in 65 participants (18.4%) or 7.49 per 1000 person-week. During the follow up period 12.7% died among which 44.7% had DILI and only 5% had severe DILI before they died but none of the deaths in our cohort were secondary to liver failure. From the total of 65 patients who developed severe DILI 26 (40.4%) were taking D4T30/3TC/EFV (Table 1), There was no significant differences in the incidence of DILI between patients who received D4T and those who did not.

### Pharmacokinetic and biochemical predictors of DILI

**Figure 1:**
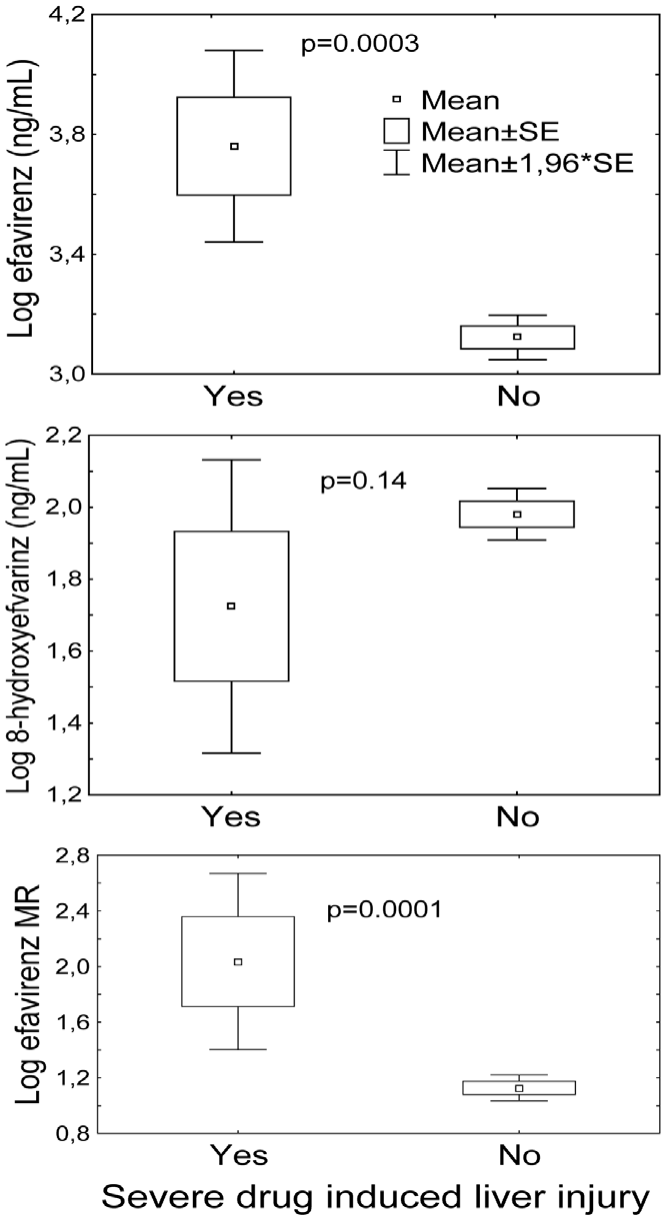
Comparison efavirenz pharmacokinetics between DILI cases and controls: mean log transformed plasma efavirenz (ng/mL). 8-hydroxyefavirenz (ng/mL) and efavirenz/8-hydroxyefavirenz ratio (MR) was compared between patients who developed concomitant efavirenz-based HAART and rifampicin based anti-tubercular drugs induced liver injury (cases) and those who did not (controls).

Analysis of baseline biochemical characteristics and efavirenz kinetics with development of DILI is presented in Table 2. There was a statistically significant association between DILI and female sex, having lower baseline hemoglobin, lower albumin, elevated baseline AST and ALT level, increased plasma efavirenz concentration and efavirenz/8-OH efavirenz metabolic ratio with p values of 0.001, 0.008, 0.007, 0.022, 0.014, 0.009, and 0.036, respectively. Comparison of mean log plasma efavirenz, 8-hydroxyefavirenz and efavirenz/8-hydroxyefavirenz ratio between patients who developed DILI and who did not is presented Figure 1.

**Table 2:** Comparison of median and inter quartile range of pre-treatment biochemical variables, liver chemistry tests and efavirenz kinetics between TB/HIV co-infected patients who developed anti-TB and efavirenz based HAART induced sever liver injury and who did not using Cox regression analysis.

Parameters	Anti TB and EFV based ART DILI		P	Exp(ß)	95.0% CI for Exp(ß)	
	Yes	No			Lower	Upper
Log EFV (ng/mL)	3.42 (3.04–3.71)	3.12(2.99–3.30)	0.004	4.568	1.610	12.965
Log 8-OH EFV (ng/mL)	2.00 (1.79–2.16)	1.95 (1.79–2.25)	0.65	0.768	0.240	2.453
Log EFV MR	1.44 (1.02–1.95)	1.17 (0.88–1.49)	0.012	2.45	1.214	4.954
Hemoglobin	10.8 (9.4–11.7)	11.5 (10.0–12.9)	0.008	0.877	0.796	9.666
AST (U/L)	71.0 (39–110)	40.0 (32–61)	0.022	1.012	1.002	1.023
ALT (U/L)	62.0 (39.0–111)	42 (32–61)	0.014	1.011	1.002	1.020
ALP (U/L)	158 (110–240)	116 (91–159)	0.124	1.002	0.999	1.004
total bilirubin (µmol/L)	0.49 (.30–.89)	0.46 (.31–.87)	0.972	0.991	0.609	1.614
direct bilirubin (µmol/L)	0.05 (.05–.24)	0.05 (0.5–.19)	0.182	2.456	0.657	9.180
Serum albumin	3.4 (2.9–3.9)	3.7 (3.2–4.1)	0.007	0.666	0.494	0.897
Urea	25.0 (20.0–31.0)	27 (20–33)	0.540	0.995	0.978	1.012
serum creatinine µmol/L	0.90 (.70–1.05)	0.9 (.8–1.1)	0.374	0.675	0.284	1.606
CD4 count/mL	75 (47–127)	96 (50–137)	0.073	0.996	0.992	1.000
Log plasma viral load	5.12 (4.26–5.64)	5.03 (4.51–5.50)	0.397	1.137	0.845	1.531

### NAT-2, CYP2B6, CYP3A5, ABCB1, UGT2B7*2, and SLCO1B1 genotype and development of DILI

Out of the 353 participants who were included in the association analysis, genotyping for NAT-2, CYP2B6, CYP3A5, ABCB1, UGT2B7*2, and SLCO1B1 genotype was done for 201. Association of each genotype with development of sever DILI is presented in Table 3. Frequency distribution of NAT2 genotype, alleles and deduced phenotype between DILI cases and controls is presented in Table 4. *NAT2* haplotypes were determined using Haploview following the nomenclature described in http://louisville.edu/medschool/pharmacology/NAT.html. According to the *NAT2* genotypes, all participants were stratified into rapid (carrier of NAT2*4, *12 or *13) and slow (homozygous for the defective variant allele NAT2*5, *6 or *7 or combination there off). *NAT2* genotypic analysis of the different SNPs showed that 138 (68.7%) patients were slow acetylators, while 63 (31.3%) were rapid acetylators. *NAT2* rapid acetylator genotype was a significantly reduced the risk of developing DILI. None of the patients who developed DILI were homozygous for functional *NAT2* variant allele (p<0.05). Cox-regression analysis showed that there was a statistically significant association DILI with CYP2B6*6 and *ABCB1 3435TT* genotype (Table 3). There was significant difference in the proportion of subjects with *ABCB1 3435TT* genotype between cases and controls being higher in those who developed DILI. A nearly significant effect of UGT2B7*2/*2 genotype with DILI was noted.

**Table 3:** Association of *CYP2B6*, *CYP3A5*, *NAT2*, *UGT2B7*, *SLCO1B1* and ABCB1 genotype/haplotype genes with development of concomitant anti-TB and efavirenz based ART induced liver injury using cox regression analysis.

*Genotype*	Anti TB and EFV based ART DILI		*P*	*Exp(B)*	*95.0% CI for Exp(B)*	
	Cases (n = 41)	Controls (n = 160)			Lower	Upper
*CYP2B6 516 G>T (* * *6)*						
GG	16 (38.7%)	79 (49.6%)				
GT	19 (46.8%)	66 (41.0%)	0.07	2.339	0.917	5.969
TT	6 (14.5%)	15 (9.4%)	0.04	2.054	1.045	4.037
*ABCB1 3435C>T*						
CC	29 (70.7%)	99 (61.9%)				
CT	7 (17.1%)	55 (34.4%)	0.38	0.593	0.183	1.928
TT	5 (12.2%)	6 (3.8%)	0.02	5.276	1.210	22.998
Number of *CYP3A5* * *1*						
Two	2 (4.9%)	9 (5.6%)				
0ne	17 (41.5%)	50 (31.3%)	0.67	0.673	0.317	5.936
Zero	22 (53.7%)	101 (63.1%)	0.93	0.944	0.222	4.014
*NAT2* acetylators*						
Slow	31 (75.6%)	107 (66.9%)				
Rapid	10 (24.4%)	53 (33.1%)	0.039	0.377	0.15	0.95
*UGT2B7 -372G>A*						
GG	5 (12.2%)	36 (22,5%)				
GA	22 (53.7%)	86 (53.8%)	0.26	1.735	0.660	6.762
AA	14 (34.1%)	38 (23.8%)	0.08	1.735	0.657	4.583
*SLCO1B1 388A>G (* * *1b)*						
GG	15 (36.6%)	53 (33.1%)				
AG	17 (41.5%)	87 (54.4%)	0.74	.891	0.450	1.765
AA	9 (22.0%)	20 (12.5%)	0.38	1.464	0.0617	3.474
*SLCO1B1 521T>C (* * *5)*						
TT	27 (65.9%)	107 (66.9%)				
TC	13 (31.7%)	49 (30.6%)	0.67	1.153	0.599	2.220
CC	1 (2.4%)	4 (2.5%)	0.92	.901	0.116	7.020

*see table 4 for detail NAT-2 allele and genotype frequency distribution.

Contrast analysis within each genotype group was done using one of the genotype as indicator reference. For the *NAT2* genotypes, subjects were stratified into rapid (carrier of NAT2*4, *12 or *13) and slow (homozygous for NAT2*5, *6, *7 or combination there off) acetylators.

**Table 4:** Frequency distribution of *NAT2* genotype and alleles and deduced phenotype (according to the NAT-2 nomenclature; http://louisville.edu/medschool/pharmacology/NAT2.html) between patients who developed concomitant efavirenz based ARV and rifampicin based anti-tuberculosis drug induced liver injury (DILI Yes) and who did not (DILI No).

NAT2 deduced phenotype	*NAT2* genotype/allele	DILI		Total
		No	Yes	
	***NAT2*** ** genotype**			
Rapid	*4/*4	2 (1.3%)	0	2
	*4/*12	2 (1.3%)	0	2
	*4/*13	2 (1.3%)	0	2
	*12/*12	3 (1.9%)	0	3
	*13/*13	1 (0.6%)	0	1
	*4/*5	6 (3.8%)	2 (4.9%)	8
	*4/*6	11 (6.9%)	2 (4.9%)	13
	*5/*12	15 (9.4%)	3 (7.3%)	17
	*5/*13	1 (0.6%)	1 (2.4%)	2
	*6/*12	9 (5.6%)	0 (0)	9
	*6/*13	1 (0.6%)	2 (4.9%)	3
Slow	*5/*5	33 (20.6%)	9 (22.0%)	43
	*5/*6	39 (24.4%)	13 (31.7%)	52
	*5/*7	8 (5.0%)	1 (2.4%)	9
	*6/*6	23 (14.4%)	7 (17.1%)	30
	*6/*7	4 (2.5%)	1 (2.4%)	5
	***NAT2*** ** alleles**			
Rapid	*4 (reference)	7,80%	4,90%	7,20%
	*12 (*803A>G*, rs1208)	10,00%	3,70%	8,50%
	*13 (*282C>T*, rs1041983)	1,90%	3,70%	2,20%
Slow	*5 (341T>C,rs1801280)	42,20%	46,30%	43,30%
	*6 (*590G>A*, rs1799930)	34,40%	39,00%	35,30%
	*7 (*857G>A*, rs1799931)	3,80%	2,40%	3,50%

## Discussion

We performed a prospective case-control association study to identify incidence, and potential biochemical, pharmacokinetic and pharmacogenetic biomarkers for concomitant ARV and anti-TB drugs induced liver injury in TB-HIV co-infected patients receiving efavirenz based for anti retroviral and RIF based anti-TB drugs. We found a higher incidence of ARV and anti-TB DILI, with the median time to event being two weeks after initiation of anti-TB therapy. The result indicates association of elevated serum efavirenz plasma concentration, baseline AST and ALT level, lower baseline hemoglobin and albumin level with DILI. Pharmacogenetic analysis for common functional variant alleles in six relevant drug metabolizing and/or transporter genes namely *NAT2, CYP2B6, CYP3A5, ABCB1, UGT2B7, and SLCO1B1* were done. ABCB1 3435TT, CYP2B6*6/*6 and slow *NAT2* acetylators genotypes were identified as pharmacogenetic biomarkers for the development of ARV and anti-TB DILI in Ethiopian TB-HIV co-infected patients. To our knowledge this is the first study to extensively examine effects of pharmacogenetic variations in several relevant genes coding for ARV and anti-TB drugs metabolizing enzymes and transporter proteins as well as to investigate impact of between patient variability in systemic efavirenz exposure and baseline biochemical parameters on risk for development of DILI with a longer follow-up period.

The incidence of DILI in the present study is higher than what we reported previously for anti-TB alone [39] or EFV based ART alone in Ethiopians patients [29]. Our result is in agreement with the previous reports describing concomitant ART and anti-TB therapy exacerbates the incidence of DILI [12], [13], [40]–[42]. Given the fact that HIV and TB therapy consist of cocktail of drugs with potential drug-drug interactions, the adverse events profile of each drug could be modified or adds up during concomitant therapy.

In general, drugs used to treat HIV and TB infections are known to induce drug-metabolizing enzymes and transporter proteins. Induction might also lead to increased production of deleterious reactive intermediates and reactive oxygen species. Mechanism for combined anti-retroviral and anti-TB DILI remains elusive and is beyond the scope of the present study. Our finding indicates high efavirenz level as a possible biomarker and risk factor for DILI. RIF induces *CYP2B6* and *CYP3A* lowering EFV plasma concentration [43]. Consequently concomitant RIF therapy should lower the risk for EFV induced liver injury. Our previous [29] and present study indicate the association of higher EFV plasma concentration with DILI regardless of concomitant RIF based anti-TB therapy, whereas no association of the metabolite (8-hydroxyefvairenz) was observed (Figure 1). However concomitant rifampicin administration has not been shown to constantly reduce efavirenz concentration [44]–[46]. Alternatively inhibition of CYP enzymes by isoniazid could modulate the inducing effect of RIF [32]. Nevertheless, we noted significant association between having higher plasma efavirenz concentration and DILI not only in absence but also in the presence of rifampicin base anti TB therapy as well.

The high efavirenz plasma level in patients who developed DILI might be the result of impaired efavirenz metabolism due to liver injury caused by other factors. Alternatively direct liver toxicity by higher efavirenz plasma concentration could be a possible mechanism for efavirenz-based HAART induced liver injury in HIV patients. Association of DILI with CYP2B6*6 and UGT2B7*2, variant alleles associated with increased efavirenz plasma concentration, [19], [47] supports the later argument. In line with this, there is evidence that efavirenz reduces cellular proliferation and triggers apoptosis *in vitro*. Clinically relevant concentration of EFV is shown to be mitotoxic in human hepatic cells in a concentration-dependent manner, pertinent to direct efavirenz induced hepatotoxicity [48]. A recent study reported that increased efavirenz level exceeding a certain threshold of mitochondrial dysfunction is associated with an autophagic overload or stress and may constitute a new mechanism implicated in the pathogenesis efavirenz induced liver damage. [49].

Isoniazid is inactivated by *NAT2* in the liver resulting in acetylisoniazid, which is further hydrolyzed to monoacetylhydrazine (MAH) [50]. Earlier studies suggested that fast acetylators were at higher risk for liver injury because they generated more acetyl-isoniazid, which could be further metabolized to other toxic intermediaries [51], [52]. However, fast acetylators clear MAH more rapidly and hence slow acetylators may have greater cumulative MAH exposure. Increased susceptibility to DILI among slow acetylators [53]–[56] or a lack of correlation with acetylation rate has been reported [57]–[59]. We found that slow acetylation status is the predominant phenotype in Ethiopian TB-HIV co-infected patients as the majorities (68%) of patients were homozygous for the defective variant allele. Therefore we classified patients having at least one functional variant allele as rapid acetylator genotype group. Interestingly we found having the rapid acetylator genotype significantly lowers the risk for DILI. Our finding is in line with the recent report where HIV-positive patients that have the slow acetylation profile are significantly associated with a higher risk of developing liver toxicity due to anti-TB drugs [56].

Several anti TB drugs and antiretroviral drugs and are substrates of P-glycoprotein, coded by ABCB1 gene. We found association of *ABCB1 3435TT* genotype with increased risk for development of DILI. Interestingly the proportion of patients homozygous for *ABCB1 3435TT* genotype was three fold higher in the patients who developed DILI compare to those who did not. *ABCB1* is responsible for the transport of many antiretroviral and anti tuberculosis drugs including rifampicin and ethambutol. *ABCB1 3435T* variant allele is reported to lower expression level and protein folding thereby altering the structure of substrate binding sites to and decreased transport activity [60]. Result from the present study indicates that this SNP may be associated with predisposition to ART and rifampicin based anti-TB DILI through a possible low transport activity.

Apart from pharmacogenetic and pharmacokinetic predictors, our result indicates a strong association between development of DILI and baseline elevation in serum aminotransferases as seen in other studies indicating that pretreatment liver enzymes are good predictors for development of DILI. The median time for development of DILI was two weeks, and 91.6% occurred during the first 8 weeks of follow up. This finding has clinical importance in providing information when to frequently assess this group of patients for development of DILI. Five of the patients who discontinued treatment because of severe DILI restarted their anti-TB treatment successfully (none discontinued HAART).

In summary, we report higher incidence of concomitant anti-tubercular and efavirenz based ARV DILI among Ethiopian TB-HIV co-infected patient The identified predictors include; slow acetylation status, CYP2B6*6/*6 and *ABCB13435TT* genotype, elevated baseline liver aminotransferases, high plasma efavirenz concentration, lower hemoglobin and albumin levels. Our result demonstrates that inter-patient variability in systemic efavirenz exposure and pharmacogenetic variation in *NAT2, CYP2B6 and ABCB1* gene determines susceptibility to efavirenz induced liver injury in HIV patients and patients. Close follow up and regular monitoring of plasma efavirenz concentration and liver enzymes during early therapy is recommended, particularly in patients with the described underlying risk factors for early diagnosis and management of efavirenz-based HAART induced liver injury. Therapeutic drug monitoring may not be feasible in resource limited setting. Therefore genotyping practice for common functional variants of *NAT2, CYP3A5 and CYP2B6* gene in HIV clinics before initiation of therapy is recommended to identify susceptible individuals and optimize the safety of concomitant rifampicin based anti-TB and efavirenz based antiretroviral therapy in co-infected patients.
